# The role of community participation in primary health care: practices of South African health committees

**DOI:** 10.1017/S146342362100027X

**Published:** 2021-06-15

**Authors:** Hanne Jensen Haricharan, Maria Stuttaford, Leslie London

**Affiliations:** School of Public Health and Family Medicine, Faculty of Health Sciences, University of Cape Town, Cape Town, South Africa

**Keywords:** community participation, health committees, human rights, primary health care, South Africa, Universal Health Coverage

## Abstract

**Background:**

Community participation is an essential component in a primary health care (PHC) and a human rights approach to health. In South Africa, community participation in PHC is organised through health committees linked to all clinics.

**Aims:**

This paper analyses health committees’ roles, their degree of influence in decision-making and factors impacting their participation.

**Methods:**

Data were collected through a mixed-methods study consisting of a cross-sectional survey, focus groups, interviews and observations. The findings from the survey were analysed using simple descriptive statistics. The qualitative data were analysed using thematic content analysis. Data on health committees’ roles were analysed according to a conceptual framework adapted from the Arnstein ladder of participation to measure the degree of participation.

**Findings:**

The study found that 55 per cent of clinics in Cape Town were linked to a health committee. The existing health committees faced sustainability and functionality challenges and primarily practised a form of limited participation. Their decision-making influence was curtailed, and they mainly functioned as a voluntary workforce assisting clinics with health promotion talks and day-to-day operational tasks. Several factors impacted health committee participation, including lack of clarity on health committees’ roles, health committee members’ skills, attitudes of facility managers and ward councillors, limited resources and support and lack of recognition.

**Conclusions:**

To create meaningful participation, health committee roles should be defined in accordance with a PHC and human rights framework. Their primary role should be to function as health governance structures at facility level, but they should also have access to influence policy development. Consideration should be given to their potential involvement in addressing social determinants of health. Effective participation requires an enabling environment, including support, financial resources and training.

## Introduction

Community participation has been viewed as a central part of the primary health care (PHC)^1^ approach since the Alma-Ata Declaration, which emphasises participation in planning and implementing health care (World Health Organization, 1978). With the signing of the Astana Declaration (World Health Organization, 2018) to mark the 40^th^ anniversary of the Alma-Ata Declaration, member states confirmed their commitments to participation. Furthermore, participation is an essential feature in the WHO’s Framework on Integrated, People-centred Health Services (World Health Organization, 2016), which frames social participation as a way of strengthening health governance.

Participation is also a cornerstone in a human rights framework. General Comment 14 on the Right to the Highest Attainable Standard of Health (UN Committee on Economic, Social and Cultural Rights, 2000) positions participation as a central component of the Right to Health. The General Comment, which is an interpretation of the Right to Health,^2^ defines participation as ‘decision-making’ that should occur at local, national and international level (ibid: 14:3). Further, General Comment 14 specifies that participation entails being part of political decisions related to health (ibid: 14:5), including participation in developing a national public health strategy and action plan. Moreover, the comment emphasises that member states have an obligation to put in place mechanisms for participation (ibid: 14:12). South Africa ratified General Comment 14 in 2015 and submitted its first progress report concerning the comment in 2017 (UN Human Rights Office of the High Commissioner, 2017).

The Alma-Ata and Astana Declarations, General Comment 14 and WHO’s Framework on Integrated People-centred Health Services together provide a framework for social participation, which define participation as involvement in decision-making concerning priority setting, planning, implementation and evaluation at local, national and global levels. In other words, participation in health is about participation in health governance.

### Community participation as part of a PHC approach in South Africa

In South Africa, community participation became part of a wider ongoing health system reform post-apartheid, which aimed to move away from a centralised, mainly curative health system to establish a decentralised district health system. The notion of participation features prominently in the post-apartheid government’s pivotal health policy, the *White Paper on Transformation of the Health System* (Department of Health, 1997) – henceforth the White Paper. The White Paper describes active participation as essential to a PHC approach and conceives participation as entailing community involvement in ‘various aspects of the planning and provision of health services’ (Department of Health, 1997: ss2.5.2 (a)). It also emphasises the importance of establishing mechanisms to improve accountability and promote dialogue and feedback between the public and health care providers. Like General Comment 14, the White Paper emphasises peoples’ participation in national policy and proposes national, provincial and district health summits as public participation mechanisms (ss. 2.5.3).

Community participation in South Africa was subsequently formalised in the *National Health Act 61 of 2003* (NHA) (Department of Health, 2004), with provisions for establishing health committees, hospital boards and district health councils. The NHA stipulates that each clinic, or a cluster of clinics, should have a health committee. The committee should be composed of one or more local government councillor(s), the head(s) of the health facility/facilities and one or more community members in the area served by the health facility/facilities. Furthermore, the NHA requires that the country’s nine provincial governments develop legislation that stipulates health committees’ role and functioning. A rapid appraisal of health committee policies showed that all South Africa’s nine provinces have some form of guideline, legislation or draft legislation on health committees (Haricharan, 2015).

### National and provincial legislation

Though health committee legislation is a provincial prerogative, it is mentioned in national policy papers. A *Draft Policy Paper on Health Governance Structures,* henceforth the National Draft Policy (Department of Health, 2013), outlines a vision of health committees as governances structures with substantial roles similar to those in the White Paper. South Africa is currently restructuring its health system by introducing Universal Health Coverage through the *National Health Insurance Bill* (NHI) (Department of Health, 2019), including re-engineering PHC services. Though early versions of the NHI paper included sections on health committees, the NHI Bill of 2019 has omitted these.

In the Western Cape Province, where this research took place, a *Draft Policy Framework for Community Participation/Governance Structures in Health* was written in 2008 (Western Cape Department of Health, 2008) but was never implemented. A *Western Cape Health Facility Boards and Health Committees Act 2016* was promulgated in 2016. Henceforth, this will be referred to as the Act or the Western Cape Act.

### South Africa’s health system

South Africa has a two-tiered health system, a public health system that serves the majority (about 84 per cent) of the population. A private system serves about 16 per cent of the population, the economically advantaged sections of the population. The South African State funds public PHC, and services are free. Provincial health departments manage the public health system. Private health care is either paid for by individuals or through medical aids. Participatory structures such as health committees only pertain to the public system.

### Empirical evidence: benefits of formalised community participation

Community participation takes many forms. In most low- and middle-income countries, health committees^3^ are the predominant form. Often these committees are linked to a specific facility. There is increasing evidence that community participation can positively impact health systems, despite many barriers. McCoy and colleagues (2012) infer in a systematic review that health committees have the potential to improve health services if they are ‘designed and implemented with care’ (McCoy, Hall and Ridge, 2012:13).

Other research echoes this conclusion (Loewenson, Rusike and Zulu, 2004; Baez and Barron, 2006; Gryboski *et al.*, 2006; Padarath and Friedman, 2008; Glattstein-Young, 2010). Studies on health committees’ accountability are divergent. Flores (2016) found that health committees can effectively take on a monitoring and accountability role, while Molyneux and colleagues’ (2012) review of literature on community accountability structures found limited empirical data on their effectiveness. Still, the authors highlight the potential for such structures.

Studies also point out that participation often offers limited influence. In their review of community participation initiatives, which included health committees and other types of community participation, George and colleagues (2015) found that communities were primarily involved in health promotion interventions but less with governance issues. On a similar note, a study from Kenya found that health committees had limited participation in governance (Kessy, 2014).

Numerous factors influence health committees. South African and African studies point to lack of clarity on role and function, staff perceptions and attitudes, resources, poor linkages to communities, top-down approaches to decision-making, dominance by medical professionals, limited resources, limited capacity and skills, attitudes of health workers towards participation, little cooperation from health services and non-existent support (Kapiriri, Norheim and Heggenhougen, 2003; Boulle, 2007; Padarath and Friedman, 2008; Glattstein-Young, 2010; O’Meara *et al.*, 2011; Kessy, 2014; Kilewo and Frumence, 2015).

Brazil is an important example of a country with a different way of structuring community participation. Here participation occurs via municipal health councils and health conferences at various health system levels. At these conferences, community structures have input in policy processes (Coelho, 2007; Cornwall and Shankland, 2008).

### Understanding participation as decision-making

The international PHC and human rights frameworks’ view of participation as *influence in decision-making* resonates with many models designed to measure how much influence participants have in the decision-making process (Oakley, 1989; Pretty, 1995; Loewenson, 2000). The seminal work in this regard is Arnstein’s *A ladder of participation* (1969), which defines participation as citizen power and contains eight different steps signifying a sequential increase in participants’ power.

This paper explores South African health committees’ roles, their degree of participation and factors impacting their functioning and role. In a linked paper, we use this paper’s findings to analyse whether the *Western Cape Health Facility Boards and Committees Act* (2016) is likely to result in effective and meaningful participation.

## Methods

The study was an exploratory mixed-methods study consisting of a cross-sectional survey (questionnaire), in-depth interviews, focus group discussions and observations.

The study was conducted in three phases. The first phase aimed to gain a better understanding of health committees. Three members of the Cape Metropolitan Healthcare Forum, an umbrella body for health committees in Cape Town, were chosen for these interviews based on their extensive knowledge of health committees. We also selected three health committees for focus groups with the same aim as the interviews. The health committees were chosen to ensure that they represented diverse experiences and the different socio-economic conditions health committees operate in. A total of 24 health committee members participated in focus groups. The interviews lasted about 1 h, while the focus groups lasted between 1 and 2 h. The data from the interviews and focus groups were analysed and used to generate the questionnaire. Data from interviews, focus groups and observations were also coded thematically.

The survey was conducted between 2010 and 2012. We conducted the survey with all willing health committee members in the Cape Metropole. The first author participated in health committee meetings prior to completing the research and made observations during these meetings. During these meetings, she also had informal discussions with the committees. Both observations and informal discussions were captured in field notes. Health committee members completed the questionnaire with the guidance of the researcher where needed.

The last phase consisted of three interviews with members of health committees, which had folded. These interviews were added after a preliminary analysis of data suggested that sustainability was a critical issue. The purpose of these interviews was to deepen our understanding of the challenges that health committees faced concerning sustainability.

The survey data were captured in MS Excel. The data were post-coded and analysed using simple descriptive statistics, while open-ended survey questions were coded thematically. The data on health committees’ roles were analysed according to degree of participation, using a framework adapted from Arnstein’s model. The data from interviews, focus groups and observations were analysed manually using thematic content analysis. This research was approved by the Human Research Ethics Committee at the University of Cape Town’s Faculty of Health Sciences (179/2007).

## Findings and analysis

### Limited reach, sustainability and functionality

The research identified 62 health committees linked to 82 clinics (some health committees are linked to more than one clinic), equivalent to 55 per cent of all 149 clinics in the Cape Town Metropole. Fifty-nine health committees participated in the research, three of them in focus groups and 56 in the survey questionnaire. Two hundred forty-six questionnaires were collected from the 56 health committees, approximately four to five per health committee on average.

The existence of health committees at 55 per cent of all clinics falls short of the NHA target, which stipulates that each clinic should have a health committee. Additionally, many health committees struggled with sustainability. There were several indicators of this during the research process. Health committees would refer to previous committees that just ‘disappeared’, ‘collapsed’’ or ‘died’. The majority of these health committees were said to start with high member attendance at health committee meetings. After a few months, members stopped attending meetings, often leaving one or two members to carry on the committee if it did not cease to exist.

Sustainability was also linked to the functionality of health committees, which varied hugely. Many health committees were reported to have a constitution, meetings were held according to an agenda, and minutes were taken. In other cases, committees operated without a constitution, agendas and minutes. Frequent cancellations of meetings were also observed, as was ad hoc organisation of meetings and low attendance. It was difficult for health committees to retain members and to stay afloat. When health committees struggled to keep their membership, these committees would sometimes attempt to recruit community members to come on board.

### Limited roles

**Figure 1.**
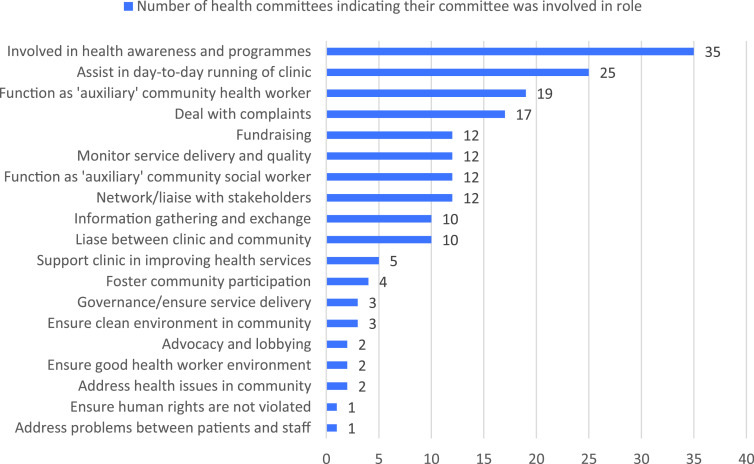
Health committees’ roles.

As Figure 1 shows, health committees played a variety of roles in the health system.

Based on the survey, the most common activity health committees indicated their committee was involved in was raising health awareness and being part of programmes at the clinic. Thirty-five health committees (62 per cent) were engaged in such activities, with the majority giving health talks at the clinic. The second most important role was assisting in the clinic’s day-to-day running, which 25 of the health committees (45 per cent) were engaged in. These activities included members functioning as security guards, cleaners or receptionists. It also included helping facility staff manage tensions in the clinic arising from patient dissatisfaction.

Nineteen committees (34 per cent) indicated that they functioned in capacities that can best be described as ‘auxiliary’ community health workers assisting the clinic with health issues such as immunisation campaigns and functioning as home-based carers. Slightly less, 17 committees (30 per cent), were involved in complaints, while 15 (27 per cent) participated in fundraising activities. Twelve committees (21 per cent) were engaged in tasks that can be described as being ‘auxiliary’ social workers taking on tasks such as helping people procure identity documents and birth certificates and running soup kitchens and feeding schemes. Twelve health committees (21 per cent) indicated that they were involved in monitoring service delivery and quality.

The roles that fewest committees were involved in was ensuring service delivery and governance, which two committees (four per cent) reported as a role. The same number of committees supported the clinic in improving health services, advocacy and lobbying, ensuring that human rights are not violated, and, finally, ensuring a good health worker environment. None reported being engaged in influencing policy or in drawing up budgets.

### Degrees of participation

Within each role, there were varying degrees of participation, defined as decision-making influence. For instance, while dealing with complaints was a relatively important role, a more detailed analysis of committees’ involvement in complaints indicated that this did not always entail being part of identifying problems or finding solutions. Instead, half of the committee members involved in complaints reported receiving, recording and handing over complaints to the facility manager. They also kept statistics on complaints but were not engaged in addressing complaints or finding solutions to issues raised. None were part of the process of redress.

To better understand health committee participation, we conducted a separate analysis of degrees of influence. A framework to assess the degree of influence in decision-making in health governance participation was developed. This framework adapts Arnstein’s framework but in a simpler version. Also, our model differs from Arnstein's in adding a category that reflects activities where community members address social determinants of health but without collaboration with health facilities. This category was included based on our empirical findings. Table 1 below outlines four degrees of participation.

**Table 1.** Degrees of participation as applied to health committees

Degree of participation	Description
Limited participation	Control and decision-making remain with the facilities. Health committees are not part of identifying problems and solutions and have limited influence.
Partial participation	Health committees are asked for input, advice or approval but have limited influence in decision-making, identifying problems and finding solutions.
Meaningful participation	Health committees are part of identifying problems, finding solutions and making decisions. Includes participation in health governance at facility level, including accountability. Participation in policy also falls under meaningful participation.
Participation in addressing social determinants of health	Participation in addressing social determinants of health.

We use the term meaningful participation to indicate involvement in decision-making. This is in accordance with a PHC and human rights approach to participation. We do not imply that activities that fall outside this category are ‘meaningless’, but rather that they fall outside this normative notion of participation.

**Figure 2.**
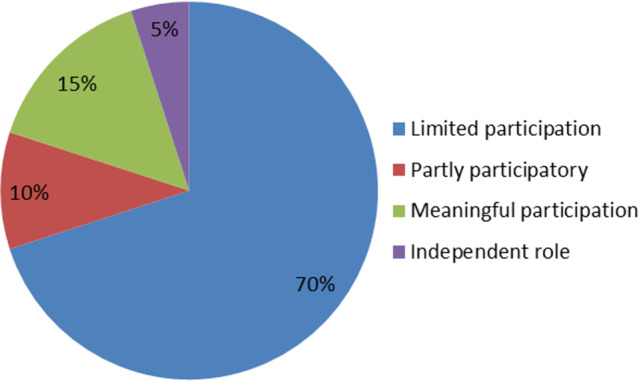
Degrees of participation.

As Figure 2 shows, 70 per cent of activities health committee members reported their committee to be involved in can be characterised as limited participation, meaning that health committee members were not part of the decision-making process. Ten per cent of reported activities were consistent with the partly participatory level. In these cases, the facility manager would ask the health committee for advice or approval. However, the health committee would not be part of setting the agenda or actively identifying issues or finding solutions.

Fifteen per cent of reported activities were characterised as meaningful participation, where health committees either planned jointly with the facility or had an oversight function. These activities included cases in which health committee members were involved in resolving complaints and addressing the issues raised in these complaints. It also included instances where health committees and facility managers together explored solutions to problems such as staff shortages, how to make the facility accessible and acceptable and improve service delivery. Five per cent of activities were directed towards addressing social determinants of health in the community, mainly addressing refuse removal. The analysis of the degree of participation is based on individual health committee members’ description.

### Lack of clarity of role and function of health committees

There were many reasons for the challenges experienced by health committees. A significant reason was that there was no legislation outlining health committees’ roles at the time of the research. This left health committees in a policy vacuum (Meier, Pardue and London, 2012).

Lack of clarity on health committee role and function was raised by committees during informal discussions and focus groups. It was also reflected in responses to the questionnaire. ‘We don’t really know what we can do’, lamented one health committee member, an expression that was reflected in many similar comments. Many health committee members answered that they needed clarity on their role and function to a survey question about what they required to function well. Confusion around their role was also reflected in the fact that the most popular choice for training was role and function of health committees (80 per cent).

A clear illustration of how uncertainty about role and function impacted committees comes from a committee that disbanded a year after it was established. The former chairperson identified uncertainty about the committee’s role as a significant stumbling block leading to limited commitment among members.

*I don’t think the people that joined the health committee knew what was expected of them. And I myself – I mean, as I said to you earlier, I myself didn’t know. That’s why when we had this meeting with the senior people from Cape Town, that’s why I asked for assistance. As I said to you, I was not geared up as to how about running this health committee, and I needed some assistance from them to guide me as to what to do and how to do it. And, of course, that wasn’t forthcoming. So, I couldn’t relate to people very well and tell them what to do and how to do it if I didn’t know myself.*

### Limited skills

Limited skills were repeatedly mentioned as a critical factor for health committees’ low functioning and limited role. When health committee members were asked to specify which skills they possessed, their responses revealed that they mostly had skills required to assist the clinic and function as ‘auxiliary’ community health care workers. In descending order, most members had skills in the following areas: home-based care, TB care/Direct Observed Treatment support, HIV/AIDS counselling, fundraising, complaints, first aid, health promotion and awareness, making food, being ‘eyes and ears’ of the community and cleaning. Conversely, nobody indicated that they had the skills to be involved in budgeting, lobbying, governance or policy. This suggests a correlation between the tasks, which health committees carried out, and the skills they possessed.

**Figure 3.**
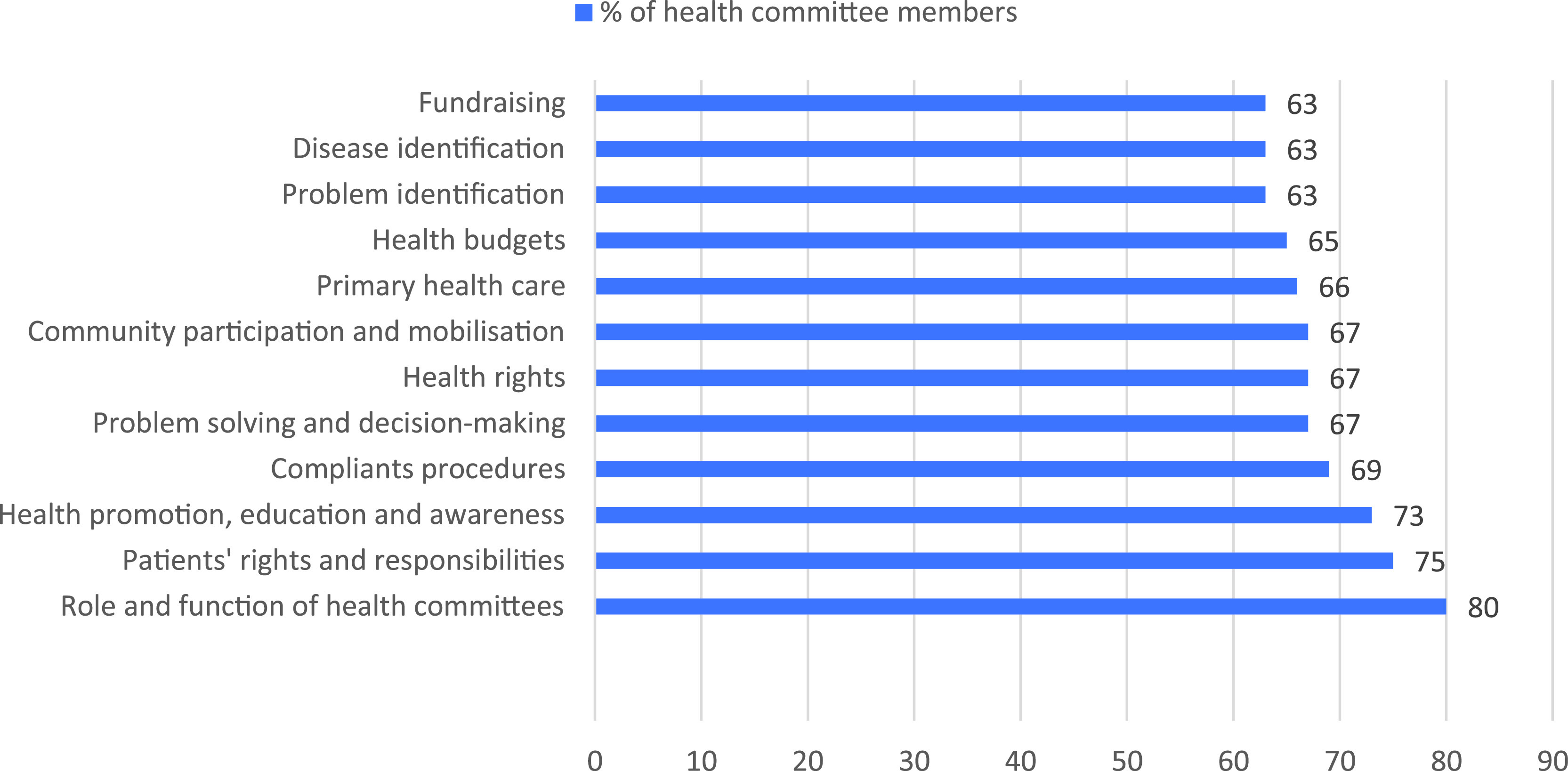
Ten most requested training topics, shown as a percentage of health committee members wanting training in the topic.

Health committee members were asked to select training topics they deemed valuable amongst a list of topics. The 10 most chosen topics, represented in Figure 3 below, provide an interesting take on how health committees see their role. One can assume that there is a correlation between training wishes and their envisioned role.

Again, it is worth noting that clarity on role and function is foremost on the list. The inclusion of topics such as community participation, budgets, complaints procedures, health rights and PHC indicates that health committee members envisioned a more expansive role than their actual role. Their training wishes could be interpreted as a signal that health committees would like to play different roles if capacitated. Thus, training should be viewed as a prerequisite for meaningful participation.

### Presence and attitude of facility managers

Relatively low attendance by facility managers at health committee meetings also impacted health committees’ functioning and limited role. The research found that facility managers were present at 44 per cent of the health committee meetings. However, they or a substitute were reportedly present ‘most of the time’ or ‘often’ in 61 per cent of committee meetings.

There were many examples of health committees in which the facility manager played a positive and enabling role. They assisted the committee in various ways, such as providing resources and access to the facility. They also made use of the health committees in multiple ways. Some committees discussed limited services and shortages of staff with the facility manager. In one health committee, meetings were used to exchange important information. The facility manager would, for example, ask the committee members to encourage women to attend screenings for cervical cancer. The discussion that followed resulted in the facility manager promising that Muslim women would be seen by a female doctor – a requirement for many Muslim women. Women from an informal settlement were also assured that they would be able to access care irrespective of whether they possessed an identity document, a standard requirement to access health care. Not all of these examples suggest that health committees participated in a decision-making process. However, they show that there was a positive collaboration with the facility, and issues related to accessibility and acceptability were addressed.

In other cases, the relationship with the facility manager was complicated or negative. There were frequent complaints about facility managers not cooperating with the health committee, ignoring its existence or refusing to share information with the committee, for instance, regarding patient complaints.

Limited cooperation with the facility manager was also crucial in two health committees that disbanded within a year. In one of the health committees, the facility manager attended only the first meeting. The chairperson commented:

*Normally, as far as the constitution indicates, the health care professional or the facility manager should be part of the health committee, and of course, we must liaise with them at all times as to what is going on. They should be invited to meetings; they should know what we need to do and participate in the functioning of the health committee. And we never got to that stage. I think they attended once, and then they just stayed away.*

In some cases, facility managers were instrumental in setting up health committees, calling meetings and setting the agenda. They functioned as the *de facto* chairperson. While this may be helpful, it also constitutes a potential problem as some facility managers took ownership of the health committee. In the absence of clearly identified roles, facility managers defined health committees’ roles. Often, the most essential role became that of assisting the clinics, filling a gap in an over-stretched health sector.

### Presence of ward councillors

Participation by ward councillors was even lower, with two (four per cent) ward councillors observed at meetings. Ward councillors were reported to attend meetings ‘rarely’ or ‘occasionally’ in 17 per cent of committees. Many committees complained that they invited the councillors but never received a response. There was a widespread perception that ward councillors were indifferent to the work of the health committees. It was evident that in the two cases where the ward councillors were present, health committees had opportunity to discuss health matters at a higher level or as a political issue. An example of this was a discussion around shortages of doctors. This resulted in a decision that the ward councillor would approach the Provincial Minister of Health to address this issue. In the other health committee, the ward councillor gave feedback on the committee’s attempts to extend services at the clinic. Again, this shows that ward councillors can assist health committees in accessing a higher health system level. In health committees without ward councillors, members often expressed frustration at not having access to higher levels of the health services or the political level with requests for annual meetings with the Provincial Minister of Health or government officials.

### Resources and support

Health committees’ poor functionality was linked to resource scarceness. Though most health committees met at the local clinic, some committees were not accommodated and had to find alternative places to hold meetings, such as libraries, police stations and community halls. For some committees, this worked, while it was a challenge for others. Access to phone, fax, computers and stationery was a problem for most committees, as was limited financial resources. Low attendance in meetings was sometimes linked to the cost of transportation and poor communication. An illustrative example is a chairperson who threatened to resign because she could not afford to pay the transport cost to attend meetings: ‘We do not even have money for transport. I have to pay out of my own pocket to go to meetings. And I can’t do it any longer. We can’t do anything because we do not have any funds’. Asked about why one of the now-defunct health committees fell apart, the former chairperson pointed to participation costs. Another health committee member noted the absence of financial resources as the main reason why the health committee never really got off the ground.

*Everybody is keen to do something – but you know with nothing, it’s not very much you can do. That’s as far as it got. Without money, you can’t do anything, and you’ll find that most people that do volunteer, they come from poor backgrounds because, I mean, basically these things are established from poor communities here – because the people that attend day hospitals come from poor communities generally. They are financially strapped, and of course, being a very bad economic climate at the moment, nobody can afford to fork out money.*

Frequently, health committees complained about not having funding for running projects as a significant problem. A disillusioned health committee chair complained that there were no funds to mark World AIDS Day, Tuberculosis (TB) month or other important days.

Another health committee member linked members’ low commitment to not being given proper resources. ‘They [the health authorities] do not meet us; they do not listen. We just get a lot of directives – health committees must do this, must do that – but no resources. People get frustrated and move to other NGOs’. Similarly, it was argued that it was difficult to attract and sustain members because of limited resources.

### Lack of recognition and political support

For some health committee members, lack of commitment was associated with a perception that health committees were not recognised and valued. This sentiment resulted in disillusionment and sometimes in disengagement from health committees. Illustrative of this is a health committee that started with 15 members but was left with five. The chairperson explained that the facility did not value the committee’s contributions. He pointed to their involvement in an event to mark a new clinic’s opening as an example. The health committee was not invited to the opening event but was asked to clean up after the event. This left the committee disillusioned, as the following quote from one of the member’s show:

*We were just there to do the dirty work, to clean up afterwards [after the opening function]. We did not know about the budget, nor did we have a chance to develop our skills. There was no discussion with the health committee about the event. It is our experience that our contributions are not valued. But you can’t just use us.*

Health committees’ frustration at not being recognised and valued was mostly directed at health facilities, but there was also little trust in political willingness to support community participation. Political support was viewed as crucial for successful participation in the following quote:

*And how to go about doing it [establish functional health committees]: you need to take it from the top shots. The Health Department’s got to get this thing on the go first of all. They’ve got to have regular meetings to tell the people what it’s all about and how they need to do these things and what assistance and guidance, to get that assistance, which they don’t do, and I think this is where they fall flat.*

### Formation of health committees

There were no clear procedures for establishing health committees. Some committees were formed by the facility manager or a community member, whom the manager asked to form a health committee. In some areas, community organisations established committees. In other cases, health committees were formed at an annual general elective meeting in communities. However, many committees explained that these meetings were poorly attended, with few people electing committee members. These formation processes resulted in weak links with the communities. Consequently, committees experienced that they were often invisible in their communities and that communities did not understand what health committees were doing. According to some health committee members, limited community interest influenced committees’ sustainability because it made it difficult to attract members and get support from communities.

Furthermore, in the cases where the facility manager played a crucial role in forming committees, health committee members were often strongly aligned with the facility. This allegiance was perhaps most clearly expressed by a chairperson who viewed health committees’ role as being that of ‘helping the staff’. The way some health committees were involved in managing queues and tensions in the clinic serves as another illustration. Some of these committees saw it as their role to manage tensions by getting patients to behave in a way deemed appropriate. They talked about how they intervened by ‘telling people to keep their mouth shut’ or ‘to tell patients to behave and show respect to the service’. As one health committee member argued, ‘The community must not complain about the poor service’.

This allegiance may be a result of committees being formed on the initiative of facility managers. But it could also be linked to health committees’ lack of formal status and power, which means that they largely rely on facility managers’ goodwill to carry out any work. They are also dependent on the facility for support as health committees have no other form of support.

## Discussion

This study highlights that despite the NHA’s provision for health committees at all clinics, they exist only in just over half of the Cape Town Metropole’s clinics. This figure is similar to the national average in Padarath and Friedman’s (2008) national survey, which found that 57 per cent of all South African clinics were linked to a health committee. Moreover, the study showed that existing health committees struggled with sustainability and functionality, impacting their effectiveness. These findings echo both South African research (Boulle, 2007; Padarath and Friedman, 2008; Glattstein-Young, 2010) and international research (McCoy, Hall and Ridge, 2012; George *et al.*, 2015).

Furthermore, the research illustrates that formalised participation via health committees in Cape Town was a limited form of participation. This contrasts with how participation is conceptualised in PHC and rights-based frameworks and scholarly literature’s definition of participation as decision-making in health governance. Other more expansive participation activities – promoting PHC, ensuring human rights, advocacy and lobbying – recorded low priority in how health committee members reported practising their roles. Instead, health committees were primarily involved in narrow participatory roles. They mainly assisted clinics with health promotion and day-to-day operational tasks and aided patients with health and social needs. Though they often practised an accountability role by being involved in complaints and monitoring services, an analysis of degrees of participation showed that they had limited influence in decision-making in relation to accountability and monitoring.

According to a human rights’ understanding, community participation requires involvement in policy development and implementation, an issue borne out in General Comment 14 and articulated in national South African policies such as the National Draft Policy. Yet, no health committee reported being engaged with policies.

The findings on roles resonate with other literature. McCoy and colleagues’ (2012) systematic review of health committees concluded that ‘Generally, there was a tendency for HFCs [health facility committees] to operate on the lower rungs of Arnstein’s ladder’ (McCoy, Hall and Ridge, 2012:9). George and colleagues’ (2015) review also found that health committees often ended up having a supportive role, while a Kenyan study highlights health committees’ limited participation in governance (Kessy, 2014). Similarly, a 2008 study in South Africa found that health committee activities did not reflect a PHC approach, including planning, priority setting and managing services (Padarath and Friedman, 2008).

The analysis of health committees’ roles shows that there were many different understandings of their roles. Developing a clear conceptual understanding of community participation and health committees’ roles is a prerequisite to effective and meaningful participation. As South Africa finalises the NHI, a re-conceptualisation of community participation in PHC is highly relevant. We argue that their roles should be viewed in relation to four domains: 1) community involvement; 2) participation in health governance at facility level; 3) addressing social determinants of health; and 4) engagement in policy.

Based on the PHC and human rights frameworks’ definition of participation, we suggest a conceptual distinction between community participation and community involvement. In contrast with participation, we define involvement as activities that do not necessarily entail decision-making; neither do these activities have to be concerned with health governance. Community involvement focuses on providing practical support to health services and communities, similar to the support health committees in the main provided at the time of the study and continue to offer in the absence of any policy interventions to focus health committee work on governance. Undoubtedly, voluntary work is an essential contribution to health services because it benefits patients and assists with significant health and social issues. However, we argue that this contribution should not be confused with participation or become a substitute for the South African State to meet its obligations to fulfil the Right to Health.

We propose that health committees should be conceptualised as structures primarily involved in health governance, including accountability, at facility level. This conceptualisation is consistent with a definition of meaningful participation as articulated in the international framework and expressed in the White Paper (Department of Health,^1997^) and the National Draft Policy (Department of Health, 2013).

A separate question that should be considered is health committees’ involvement in addressing social determinants of health. In this study, health committees in Cape Town had limited roles in addressing social determinants of health. There is limited evidence in the literature that health committees engage in underlying factors for ill health. Addressing social determinants in health is not articulated directly in the international frameworks or any South African legislation. This paper suggests that community participation structures’ role in addressing social determinants of health should be considered in policy, practice, PHC and human rights frameworks.

Finally, the policy context should be considered. Health committees are likely to encounter issues relevant to policies. Therefore, it would be beneficial for health committees to have access to raise issues upstream in the health system. The research showed minimal involvement in higher-level or system issues. This may be because very few ward councillors attended health committee meetings and due to the absence of structural links to facilitate upstream influence.

An effective way of ensuring influence in policy could be a tiered model where health committees are organised in broader structures that could articulate policy issues upward. In South Africa, there is no articulation between different community participation structures, such as health committees, hospital boards or district health councils. There are no other provisions for community-level representation to find its way up the decision-making hierarchies in South Africa’s health system to provincial and national levels.

A tiered model for participation could resemble Brazil’s model of social participation in health, which occur via public health conferences at different levels from the very local level to the national level. These conferences are noted to raise many policy issues (Cornwall and Shankland, 2008). It is also worth noting that Backman and colleagues (Backman *et al.*, 2008) point out that one of the indicators to understand the extent to which health systems are consistent with the Right to Health is that a national health plan is developed with meaningful input from communities.

Our study also calls for attention to facility managers and ward councillors’ participation in committee meetings. Considering how facility managers’ participation in health committee meetings could be enforced, for instance, by making it part of their key performance area, would be significant. Further, facility managers’ attitude to participatory structures is imperative to relook. Zwama (2017) noted in an evaluation of health worker training that this training influenced facility managers and health workers’ understanding of participation and intention towards collaboration. This could be viewed as an argument for the importance of capacitating facility managers to engage in community participation. However, there is also a need to explore facility managers’ attitudes to community participation.

Similarly, ways of ensuring ward councillor participation should be explored. Ward councillors’ lack of interest in health committees could be interpreted as an expression of lacking political will to ensure meaningful community participation. But it could also be seen as a reflection of the institutional arrangements where no legislation provides for an influential role for committees, and health committees do not have any formal power. Currently, ward councillors represent the only link between health committees and the political system. Research that understands their perceptions of health committee participation would be valuable to understand their limited participation.

The paper also highlights issues related to how health committees are formed: through a process where the facility manager is in charge or through a process led by community members. This study has not provided sufficient data to suggest a specific approach. It has, however, demonstrated that the formation process influences how health committees represent community needs. This should be explored further.

### Creating enabling conditions

The study underscores that effective participation requires an enabling environment. Part of an enabling environment is supportive facility managers and ward councillors. But – as emphasised by health committee members – political support is also essential. In addition, committees also need access to a venue, resources, support and training. The research demonstrated how limited financial resources resulted in poorly functioning committees that could not initiate their own projects. Having to bear the ‘cost of participation’ places an undue burden on people, often living in depressed socio-economic conditions. It resulted in frustration and sometimes in disengagement. Moreover, limited funding also had a negative symbolic value, as it was perceived to signal a lack of recognition. Several studies have documented similar detrimental impact of lack of resources, skills and support (Boulle, 2007; Padarath and Friedman, 2008; Glattstein-Young, 2010).

### Legislation

Researchers and practitioners from Eastern and Southern Africa have called for a legislative mandate for health committees (Equinet, 2014). This research has highlighted that without legislation, health committee participation is likely to be a form of limited participation, inconsistent with the vision outlined in the international PHC and human rights framework. Further, committees are likely to struggle with reach, functionality and sustainability without an appropriate legislative framework which ensures sufficient support.

### Study limitations

One drawback of this study is that the data were collected in 2010–2012. However, our experience from our continued work with health committees, confirmed in the discussions at two national colloquia on health committees in South Africa in 2014 (Mdaka, Haricharan and London, 2014) and 2017 (Naidoo, 2017), is that the findings of this study are consistent over time and across provinces, both in terms of limited roles and the challenges health committees face. Therefore, the analysis is still highly relevant to understanding health committees’ roles and their challenges.

Furthermore, we do not know the size of health committees – partly because membership was sometimes a fluid concept. Hence, it is difficult to give a figure for the percentage of health committee members who participated in the study.

## Conclusion

This study found that health committees exist only at just over half of PHC clinics in the Cape Town metro despite a legal requirement in the NHA that all clinics have a health committee. Existing health committees struggle with functionality and sustainability. Furthermore, health committees played a limited role. Their primary roles focused on supporting the clinic while they had a limited role in health governance. To a large degree, their roles were inconsistent with a meaningful form of participation, which entails decision-making. Few health committees were concerned with social determinants of health, and none influenced policy. The paper also identified several factors impacting health committees, including lack of clarity on roles, health committee members’ skills, presence and attitude of facility managers and ward councillors, limited resources, support and lack of recognition.

The paper argues that a meaningful form for participation could entail that health committees are defined as governance structures at facility level. Health committees could also have substantial roles in addressing social determinants of health. They should have access to address issues at the policy level either directly or through a tiered community participation system. Effective and meaningful participation requires legislation that clearly outlines health committees’ roles and ensures an enabling and supportive environment. This includes facility managers and ward councillors who participate in health committee meetings, training health committees, resources and support.

## Footnotes

1 When we talk about PHC, Primary Health Care, we refer to an approach to the health system formulated in the Alma-Ata Declaration. We differentiate this from primary care or primary health care (lower case), which refers to health services at the primary level. When we write about the international PHC and human rights framework, it refers to the Alma-Ata and Astana Declarations and General Comment 14.

2 A General Comment is a treaty body’s interpretation of human rights treaty provisions, thematic issues or its work methods. General comments often seek to clarify the reporting duties of State parties concerning specific provisions and suggest approaches to implementing treaty provisions.

3 Health committees are sometimes referred to as clinic committees or health facility committees. The terms are used interchangeably in this paper, though we most frequently use the term health committees.
